# Spectral Failsafe System of High-Power Laser Using Dual Fiber Bragg Gratings

**DOI:** 10.3390/mi14101927

**Published:** 2023-10-14

**Authors:** Zhaoyu Zong, Xiaocheng Tian, Mengqiu Fan, Dandan Zhou, Rui Zhang, Junpu Zhao, Wanguo Zheng, Dangpeng Xu

**Affiliations:** Laser Fusion Research Center, China Academy of Engineering Physics, Mianyang 621900, China; zongzhaoyu1st@163.com (Z.Z.); tianxc203@126.com (X.T.); fanmengqiu@163.com (M.F.); dan723@126.com (D.Z.); zhangrui8s-1@caep.cn (R.Z.); lfrczhaojunpu@caep.cn (J.Z.); wgzheng_caep@sina.com (W.Z.)

**Keywords:** high-power laser, transverse stimulated Brillouin scattering, phase modulation, failsafe system, fiber Bragg gratings

## Abstract

Phase-modulated (PM) spectral failsafe systems are necessary to promptly terminate amplification processes following accidental seeding of a high-power laser chain with a non-PM pulse to prevent optical damage. In this work, we present a reliable spectral failsafe system that can indicate the presence or absence of sufficient PM light. This requirement is met by combining dual temperature-sensitive fiber Bragg gratings detection with high-speed RF amplitude comparisons. The failsafe trigger signal is generated when the spectral power at the peak sideband exceeds that at the center. The spectral failsafe system has the ability to distinguish between adequate and inadequate PM pulses, and it exhibits significant robustness in pulse width, TEC temperature drift, and DFB wavelength drift in experiments, making it valuable for safe high-power laser operations and providing a useful reference for other detection system designs.

## 1. Introduction

High-power lasers are excellent tools for investigating high-density physics, high-intensity interactions between light and matter, and plasma physics [1,2,3]. One far-reaching potential application of high-power lasers is inertial confinement fusion (ICF), where nuclear fusion reactions are initiated by heating and compressing a deuterium–tritium (DT) target with high-power nanosecond lasers [4,5]. Large-scale high-power laser facilities designed as ICF drivers have been developed rapidly in the last decades, such as the National Ignition Facility (NIF) at Lawrence Livermore National Laboratory (LLNL) in America [6,7], the Laser Megajoule (LMJ) in France [8], and the SG-series laser facility in China [3,9].

With the goal of delivering as much energy as possible to the target, the ICF drivers are faced with conditions reaching the threshold for transverse stimulated Brillouin scattering (TSBS), a nonlinear process that is usually observed in high energy, large aperture laser systems [10,11,12]. In TSBS, a high-intensity monochromatic beam can create acoustic waves in a physical medium (such as a fused silica lens) that will scatter the beam, resulting in instability, losses, and optical damage. Even without damage, TSBS energy losses will prevent the delivery of the full energy to the target, rendering a given laser system less effective.

As a detrimental nonlinear effect in high-power large aperture laser systems, TSBS can be suppressed by adding multiple frequency sidebands on the laser pulse using an electro-optic phase modulator [13,14,15]. The basic concept of phase modulation (PM) is to distribute the energy from a single frequency laser that may exceed the TSBS threshold to numerous nearby frequency lobes with individual amplitudes that are below the TSBS power threshold. Based on the nature of the TSBS process, these lobes do not interact constructively with each other as long as the frequency spacing is greater than the reciprocal of the SBS decay time. This approach, known as TSBS suppression, has been successfully implemented at various high-power laser facilities, with modulation frequencies of 3 GHz, 2 GHz, and 14.8 GHz being used for the NIF, LMJ, and the Z-Beamlet Laser (ZBL), respectively [16,17]. In particular, we have chosen to use a modulation frequency of 2.5 GHz with a modulation index of approximately 8 radians, which results in approximately 16 sidebands within a bandwidth of approximately 40 GHz for TSBS suppression. However, there is a risk of accidentally seeding the main amplifiers with a non-PM pulse. If the seed pulse that provides PM light fails in its phase modulation and instead provides single-frequency light, all of the TSBS risks are present. Therefore, it is necessary to monitor whether there is sufficient PM light present using a reliable spectral failsafe system that will interrupt the amplification process following a failure of the PM system.

Given the importance of mitigating TSBS buildup in large optics, multiple spectral failsafe systems have been developed for ICF-like lasers. One approach involves detecting a PM sideband utilizing a fiber Bragg grating (FBG) [18,19]. This method aims to demodulate amplitude modulation on the PM spectrum. While this approach is initially attractive because it is almost entirely fiber-based and requires little free space coupling or alignment, it suffers from stability issues and introduces the additional complication of environmental perturbation of the fiber structure. Another approach involves detecting a PM sideband using an etalon as a filter [20]. This method uses a temperature-tuned solid etalon to transmit a specific PM sideband and detects the presence of PM light. While simple, it suffers from insufficient frequency discrimination, which can lead to a false positive condition if the unmodulated carrier is transmitted. The likelihood of a false positive is low, but the existence of this risk has prevented the selection of this technique. The ZBL and LMJ both adapt the optical heterodyne detection method to detect a PM sideband [16,18]. The heterodyne method provides greater safety than some simpler failsafe methods because it has built-in redundancy that reduces the possibility of a false positive condition. However, the heterodyne method is complex and more expensive than other competing methods. Therefore, spectral failsafe methods with greater safety and lower cost are of great significance for high-power laser facilities with several tens of beamlines.

In this paper, we propose a new method to indicate the presence or absence of adequate PM light simply based on an optical power comparison using two specially designed FBGs. The spectral failsafe system can distinguish adequate PM pulses from inadequate PM pulses with a high reliability. Additionally, it demonstrates significant robustness to changes in the pulse width of the PM light, ambient temperature fluctuations, and wavelength shifts within a distributed feedback (DFB) laser source. The spectral failsafe system has the advantages of economy, durability, strong interference resistance, and strong expansibility.

## 2. Design of Phase-Modulated Spectral Failsafe System

The fiber front-end is responsible for generating the single pulse that seeds the entire high-power laser facility. The pulse is sculpted into high-contrast shapes using an electrical waveform generator, then amplified and split into tens of beam lines. Before leaving the fiber front-end, every output beam is phase modulated. By employing an additional radio frequency (RF) signal to the LiNbO_3_ electro-optic phase modulator, the refractive index of the modulator can be changed; thus, the phase of the laser can be modulated.

The incident laser pulse is given by:

(1) $$E_{in}=A_{0}\left(t\right)\exp\left(i\varphi_{0}\right),$$

where $A_{0}\left(t\right)$ is the amplitude of the pulse and $\varphi_{0}$ is the initial phase of the pulse.

For sinusoidal modulation, the output PM laser can be written as:

(2) $$E_{out}=A_{0}\left(t\right)\exp\left(iM\sin\left(2\pi f_{m}t\right)+i\varphi_{0}\right),$$

where M is the modulation index and $f_{m}$ is the modulation frequency.

As the spectral information mainly depends on the phase-modulated part, we can ignore the time part in expression (2). Thus, the output PM laser can be rewritten in the expansions of the first kind of Bessel functions [13]:

(3) $$A\left(f\right)=\sum_{k=-\infty }^{\infty }J\left(k,M\right)\cdot \delta \left(f-k\cdot f_{m}\right),$$

where f is the carrier frequency and $J\left(k,M\right)$ is the k order of the first kind Bessel function at the modulation index M.

The expression given in (3) indicates that the power spectrum of the PM light is the distribution of Bessel functions, which consists of a carrier (k=0) and a series of sidebands located at positive and negative integer multiples of the modulation frequency ($f_{m}$) with amplitudes equal to the squares of the Bessel functions. Specifically, we have chosen to use a modulation frequency of 2.5 GHz with a modulation index of approximately 8 radians, resulting in approximately 16 sidebands within a 40 GHz (0.15 nm) bandwidth to suppress TSBS in the main laser optics.

Due to the crucial importance of suppressing TSBS build-up in large optics, it is essential to incorporate the spectral failsafe system into the fiber front-end design to ensure that the seed pulses are appropriately phase-modulated at 2.5 GHz. The spectral failsafe system monitors the 40 Ghz modulation bandwidth of every pulse generated in the fiber front end at a 1 kHz rate. When the spectral bandwidth of a seed pulse exceeds the threshold bandwidth for TSBS suppression, the spectral failsafe system provides a trigger to the Pockels cell (PC) gate of the preamplifier. Subsequently, a seed pulse with an adequate spectral bandwidth is amplified to the multi-joule level and transmitted to the main amplifier system. When the spectral bandwidth of a seed pulse falls below the threshold bandwidth, the spectral failsafe system inhibits the trigger to the PC gate and raises an alarm. Therefore, the spectral failsafe system is in place to ensure that the pulse cannot propagate in the high-power laser chain unless adequate modulation has been applied to ensure TSBS suppression.

The peak gain of neodymium-doped phosphate glass operates at 1053 nm. The master oscillator wavelength is precisely tuned to within 0.005 nm of the target value of 1053.00 nm through temperature control. The oscillator is a DFB fiber laser, which is capable of generating up to 20 mW of continuous wave power within a single longitudinal mode and has a very narrow spectral linewidth of 70 kHz. The lower critical threshold bandwidth for TSBS suppression is approximately 27 GHz, corresponding to a wavelength of approximately 0.10 nm. For safety reasons, we have chosen to operate at a spectral bandwidth of 0.15 nm for the high-power laser facility.

**Figure 1:**
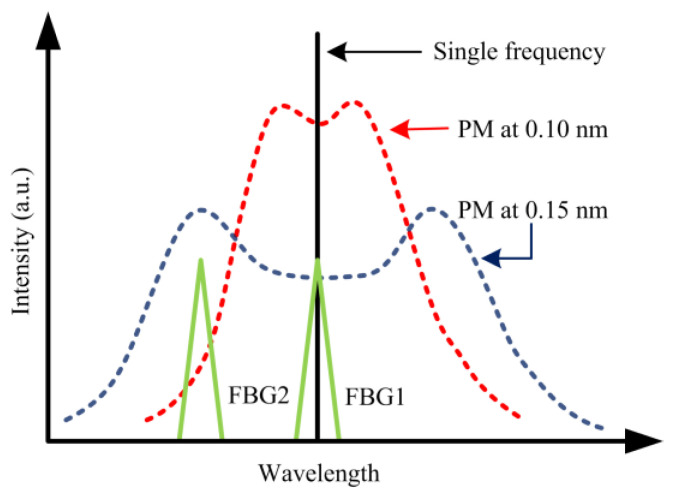
Concept of the phase-modulated spectral failsafe system.

The novel design of the PM spectral failsafe system is shown in Figure 1, where the horizontal and vertical axes represent the wavelength and normalized spectral intensity of the pulse, respectively. The black vertical line at the center of the figure represents the spectral shape of a non-PM pulse with a single frequency (0th order). The dashed red curve and dashed blue curve represent the spectral envelope shapes of PM pulses broadened to 0.10 nm and 0.15 nm bandwidths, respectively. It is evident that the power of the PM light with 0.10 nm spectral broadening is more concentrated than that of the 0.15 nm, but the power distribution at the sidebands is lower. Additionally, the green triangular lines represent the reflected spectra of narrow-band FBGs, and the reflection center can be adjusted by changing the ambient temperature using a thermoelectric cooler (TEC). As shown in Figure 1, FBG1 on the right and FBG2 on the left reflect the central band spectrum and the left peak sideband spectrum of PM light, respectively. Therefore, the presence or absence of adequate PM light can be determined by comparing the optical power in different frequency bands reflected by the FBGs. When the optical power reflected by FBG2 exceeds that reflected by FBG1, it indicates that the laser pulse is phase modulated. Conversely, when the optical power reflected by FBG2 is lower than that reflected by FBG1, it indicates that the laser pulse lacks adequate phase modulation or is a non-PM pulse.

## 3. Experimental Setup

**Figure 2:**
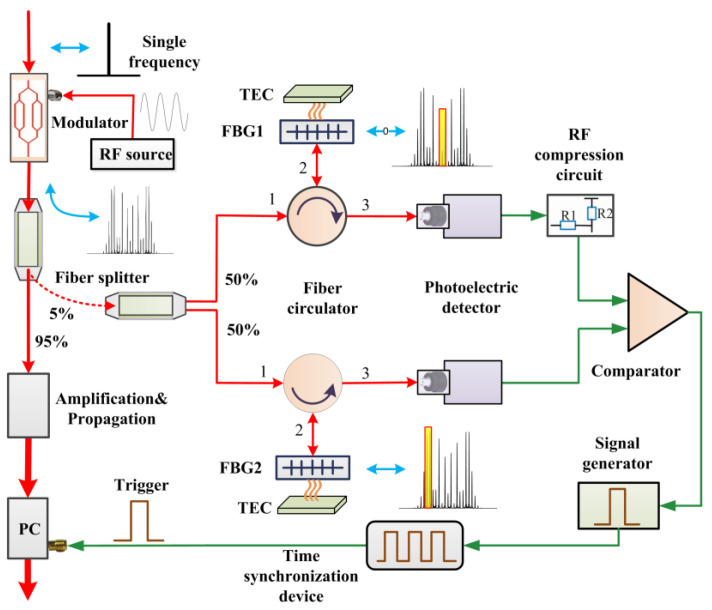
Layout of the phase-modulated spectral failsafe system.

The layout of the phase-modulated spectral failsafe system is shown in Figure 2. First, the LiNbO_3_ electro-optic modulator modulates the phase of a single-frequency laser pulse. Then, the laser pulse containing a series of frequency sidebands located at integer multiples of the modulation frequency is generated, which is known as PM light. A 95:5 fiber splitter divides the PM light into two parts, of which 95% is called the main laser signal and the remaining 5% is called the monitor light. Triggered by the failsafe interlock signal, the PC switch turns on, and the main laser signal propagates and amplifies in the preamplifier system to a multi-joule level and then injects into the main amplifier system. Note that the trigger comes from the feedback of the PM spectral detection using the remaining 5% of the light.

The monitor light, with an energy of approximately hundreds of picojoules, travels to the spectral failsafe system. The monitor light is subdivided into two equal parts by a 50:50 fiber splitter, with one half routed to the center wavelength-detecting module and the other half to the sideband wavelength-detecting module. The monitor light passes through a fiber circulator and arrives at customized narrow-band FBGs, with a reflection coefficient of 0.85. The central reflection wavelength of the FBGs can be tuned by adjusting the ambient temperature using the TEC. The optical power in different frequency bands can be extracted at the output (port 3) of the fiber circulator using the FBGs. It should be noted that FBG1 primarily reflects the central spectral bands of the PM light, while FBG2 mainly reflects the left peak sideband spectral bands of the PM light. To avoid misjudgment caused by overlapping spectral components reflected by the two FBGs, the reflection bandwidth of the FBGs should be less than the wavelength difference between the center band and the left peak band. Considering cost and performance, we select Gaussian FBGs with a reflection bandwidth of 0.05 nm. Subsequently, the selected optical power is injected into a photoelectric detector (PD) through port 3 of a fiber circulator. Specifically, an RF signal converted from the central wavelength (upper branch) arrives at the input terminal of a high-speed comparator through an RF compression circuit. The function of this RF compression circuit is to compress the RF voltages generated by the central wavelength signal, which mitigates the saturated response of the PD and enhances spectral bandwidth discrimination. Afterward, a high-speed comparator is employed to compare the RF voltages converted from the reflected light. For simplicity, the voltages converted from the FBG1 branch and the FBG2 branch are referred to as voltage-FBG1 and voltage-FBG2, respectively. When voltage-FBG1 (left peak sideband) is higher than voltage-FBG2 (center band), this indicates that the phase modulator is functioning properly, and that the laser pulse has been phase-modulated. In this case, a signal generator provides an output that serves as a failsafe trigger signal to the PC gate of a pre-amplifier in a precise time sequence via a time synchronization device. Subsequently, the main laser signal can propagate and amplify within a subsequent high-power laser chain. Conversely, when voltage-FBG2 is lower than voltage-FBG1, this indicates that the phase modulator may have failed, and that the laser pulse lacks adequate phase modulation or is a non-PM pulse. In this scenario, a signal generator inhibits the trigger to the PC gate of a pre-amplifier and reports an alarm.

We studied the performance of the key component of the spectral failsafe system. Initially, the photoelectric response characteristics of the PD were evaluated. The PD response voltage monotonically increased with laser energy injection, reaching a saturated voltage of 3.32 V at a critical laser injection level of approximately 200 pJ. Additionally, the TEC varied from 17 °C to 35 °C with an accuracy of 0.2 °C. Then, we measured the relationship between the center reflected wavelengths of the FBGs and ambient temperature by controlling the TEC. The results demonstrated that the central reflected wavelength undergoes linear red-shifting within the TEC’s adjustable range (17–35 °C), with the temperature drift coefficient amounting to 11.4 pm/°C.

The fiber-based design of the PM spectral failsafe system eliminates the need for free-space coupling or alignment, ensuring robust performance during operation. The detection scheme involves a simple comparison of optical powers and is insensitive to energy fluctuations, thereby maintaining a high reliability. As a result, despite fiber structure environmental perturbations, the spectral failsafe system remains stable and reliable with a high performance accuracy.

## 4. Results and Discussion

We studied the practical performance of the PM spectral failsafe system in the experiments. First, we precisely tuned the master oscillator wavelength to a desired value of 1053.00 nm, and the output laser pulse was phase modulated to a bandwidth of 0.15 nm. As a result, the left peak sideband wavelength of the laser pulse was approximately 1052.94 nm, while the center wavelength remained at 1053.00 nm. In this case, the ambient temperature of FBG1 was initially set to 31.5 °C, reflecting the center spectral band, and the ambient temperature of FBG2 was initially set to 26.5 °C, reflecting the left peak sideband.

We studied the availability and compatibility of the PM spectral failsafe system. Table 1 provides the characteristics of the spectral failsafe system under different laser pulse widths, where “PM” and “NO-PM” indicate that the laser pulses were phase-modulated and monochromatic, respectively. As an example, the squared-wave laser pulse with a pulse width of 3.2 ns, commonly used in HED physical experiments, is considered. As shown in Table 1, when this pulse is phased modulated, the voltage-FBG2 (2.35 V) is greater than the voltage FBG1-(1.83 V), causing the signal generator to provide a trigger output that serves as the failsafe signal to the PC gate. Conversely, when the pulse is a single-frequency light without phase modulation, the voltage- FBG2 (0 V) is lower than the voltage-FBG1 (3.32 V), causing the signal generator to inhibit the trigger to the PC gate of the pre-amplifier. Additionally, the optical delay from the output of the phase modulator to the PC gate of the pre-amplifier exceeds 500 ns, taking into account the fiber delay line and multiple rod passes in the pre-amplifier. The transition time for the failsafe trigger signal to respond to changes is approximately 45 ns, which is sufficiently short to safely stop a shot if the phase modulator faults. In other words, the maximum repetition rate limit of the PM pulse that can be reliably monitored is 22 kHz, which is significantly higher than the pulse generation frequency of 1 kHz. Therefore, when a 3.2 ns squared-wave pulse is injected into the fiber end, the PM spectral failsafe system functions correctly. Table 1 also shows that the system is compatible with a range of laser pulses that are commonly used for physical experiments in high-power laser facilities. Furthermore, the compatibility and availability of the PM spectral failsafe system were tested under different beamlines, with the results meeting the evaluation criterion. The results demonstrate that the PM spectral failsafe system has high availability for the typical temporally sculpted laser pulse.

The PM spectral failsafe system is a critical component of high-power laser facilities. To meet the reliability requirements, we further studied the critical operating conditions of the failsafe system.

**Figure 3:**
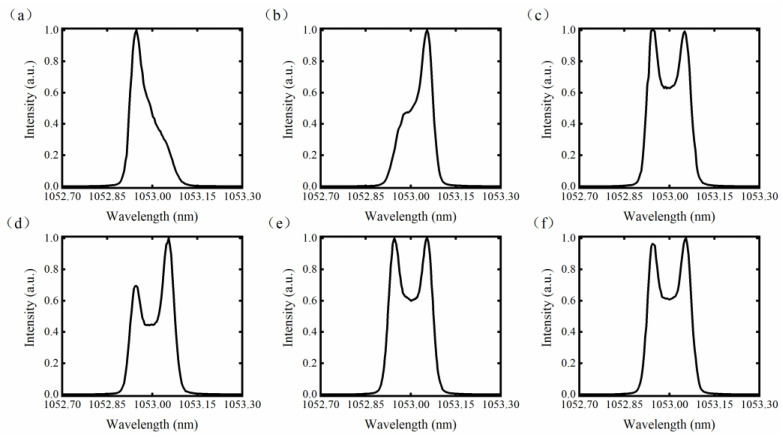
PM spectrum distribution of laser pulses with different pulse widths. (**a**) 0.3 ns; (**b**) 0.3 ns with π phase relative to (**a**); (**c**) 0.4 ns; (**d**) 0.5ns; (**e**) 3.2 ns; (**f**); 10 ns.

First, we studied the pulse width range that could be accurately characterized. Laser pulses with temporally sculpted widths ranging from 0.1 ns to 25 ns were generated using the electrical waveform generator and injected into the fiber front end. The results showed that the PM spectral state of pulse widths less than 400 ps could not be accurately characterized. To analyze the influence of pulse width on detection, the PM spectral distributions of laser pulses with different widths were subsequently measured using a fiber spectrometer, as depicted in Figure 3. Due to the limited resolution (0.04 nm) of the spectrometer, the measured PM spectral distribution presented here represents the spectral intensity envelope. Nevertheless, it is still sufficient to illustrate the correlation between the temporal shape and spectral distribution.

Figure 3a,b displays the spectral distributions of two 300 ps pulses with different initial phases, with the pulse in Figure 3a experiencing a π phase delay relative to the pulse in Figure 3b. The phase modulation frequency was 2.5 GHz, resulting in a temporal modulation period of 400 ps. When the pulse width was less than this modulation period (400 ps), both the pulse width and the initial phase of the phase modulation significantly impacted the spectral shape. In this scenario, the optical power ratio between the left peak sideband and central band is uncertain, leading to possible false judgments from the failsafe system. However, as shown in Figure 3c–f, when the pulse width exceeds or equals the modulation period, the influence of the initial phase on the spectral intensity envelope becomes negligible. The spectral distribution of the 500 ps pulse shown in Figure 3d is extremely asymmetric, with the long wavelength direction (right peak band) containing more energy, but with the intensity of the left peak band still exceeding that of the central band. In this scenario, the failsafe system can still accurately determine spectral broadening state, indicating its reliability when pulse widths are greater than or equal to the modulation periods. However, when the pulse width of the injected laser was 400 ps or less, the high-power laser facility did not perform formal shots that were close to the TSBS threshold. Therefore, although the failsafe system may make a false judgment when the pulse width is less than one modulation period, this will not affect its practical application. In addition, the failsafe system could accurately judge the PM state of the 10 ns squared wave pulse. Due to the combination of the nonlinear saturation response of the PD and the RF compression circuit, the spectral detection ability is expanded, thus realizing the compatibility for long pulse widths.

**Figure 4:**
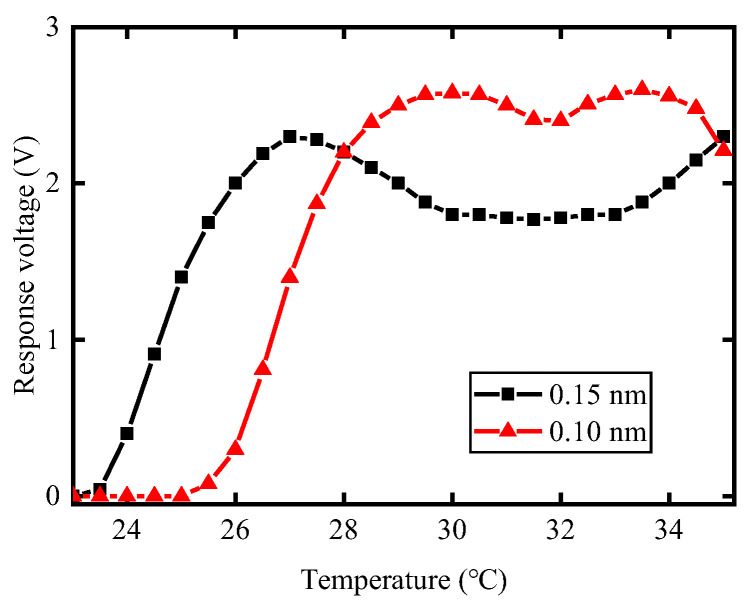
Response voltage of the phase-modulated spectral failsafe system in different temperatures.

Spectral bandwidth discrimination is one of the main indicators for quantifying the reliability of failsafe systems, representing their ability to distinguish between adequate and inadequate PM states. To provide a margin of safety, we chose 0.15 nm as the working bandwidth (adequate PM state) and 0.10 nm as the limit bandwidth (inadequate PM state) for the high-power laser facility. Figure 4 shows that the detector response voltage varied with the ambient temperature of the narrow FBG for PM bandwidths of 0.15 nm and 0.10 nm. In this measurement, different spectral components were selected by changing the ambient temperature of the narrow-band FBG. The reflection bandwidth of the narrow-band FBG was 0.05 nm, which was close to the resolution of the spectrometer (0.04 nm). The morphology of the voltage response curves obtained from FBG reflection at different ambient temperatures was similar to the spectral envelope distribution shown in Figure 3, which further indicates the practicality of the PM detection method. The voltage response range for 0.10 nm was narrower than that for 0.15 nm, but the voltage amplitude at the center band of 0.10 nm was higher than that at 0.15 nm. The voltage amplitude is related to the spectral characteristics of the pulse and the ambient temperature surrounding the FBG. Based on voltage response features, we can distinguish between different spectral broadening states of PM light and achieve accurate spectral bandwidth discrimination.

**Figure 5:**
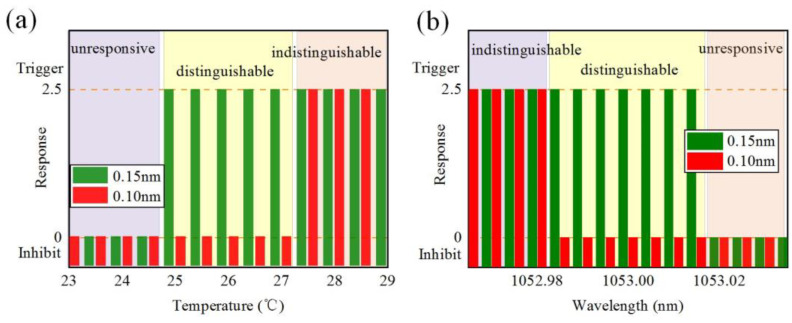
Response of the phase-modulated spectral failsafe system (**a**) under different temperatures of FBG1 and (**b**) under different wavelength drifts of the master oscillator.

Then, we studied the influence of temperature drifting of the TEC on the spectral discrimination performance. In this experiment, we kept the pulse width of the laser pulse at 3.2 ns. Additionally, we maintained the center reflection wavelength of FBG2 constant while changed the center reflection wavelength of FBG1 through ambient temperature adjustment Figure 5a shows the response of the spectral failsafe system under different ambient temperatures of FBG1. There are three different response regions in Figure 5a, including an unresponsive region, a distinguishable region, and an indistinguishable region, which are marked with light purple, light yellow, and light red, respectively. When the ambient temperature of FBG1 was in the range of 23~24.5 °C, the failsafe system did not generate a trigger signal for 0.15 nm pulse and 0.10 nm pulse. The temperature range (23~24.5 °C) is called the unresponsive region. As the ambient temperature of FBG1 rose to the range of 25~27.5 °C, the failsafe system provided a trigger signal for the 0.15 nm pulse but did not output a trigger signal for the 0.10 nm pulse. In this case, the failsafe system could distinguish between an adequate PM state (0.15 nm) and an inadequate PM state (0.10 nm). This temperature range (25~27.5 °C) is called the distinguishable region. As the ambient temperature of FBG1 rose to the range of 28~31 °C, the failsafe system provided a 2.5 V trigger signal, regardless of whether the spectrum was broadened to 0.10 nm and 0.15 nm. In other words, when the ambient temperature of FBG1 was in the range of 28~31 °C, the failsafe system could only distinguish whether the spectrum was broadened, but could not distinguish between different broadening states of the PM pulse. This temperature range (28~31 °C) is called the indistinguishable region.

As shown in Figure 4 and Figure 5a, when the ambient temperature of FBG1 is in the range of 23 °C to 24.5 °C, the spectral band (1052.90~1052.92 nm) reflected by FBG1 is far away from the center band (1053.00 nm) reflected by FBG2. For laser pulses with spectral widths of 0.15 nm and 0.10 nm, there are relatively few spectral components within this band, resulting in lower voltage-FBG1 compared to voltage-FBG2. Subsequently, the signal generator inhibits the trigger to the electro-optical gate of the preamplifier. As the ambient temperature of FBG1 rises to the range of 25 °C to 27.5 °C, the spectral band (1052.92~1052.95 nm) reflected by FBG1 undergoes a redshift, bringing it closer to the center band at 1053.00 nm. Because of the wide spectral range of the 0.15 nm pulse, there is large spectral energy in the band of 1052.92~1052.95 nm, voltage-FBG1 is greater than voltage-FBG2, and then the signal generator provides a 2.5 V trigger output that serves as the failsafe signal. However, because the spectral range of the 0.10 nm pulse is relatively narrow, there are few spectral components distributed in the same band range of 1052.92~1052.95 nm. Correspondingly, when voltage-FBG1 is smaller than voltage-FBG2 for the 0.10 nm pulse, the signal generator does not generate a trigger signal. The wide spectral range of the 0.15 nm pulse results in significant spectral energy within the band between 1052.92 nm and 1052.95 nm, leading to a higher voltage-FBG1 compared to voltage-FBG2, which then generates a 2.5 V trigger output serving as the failsafe signal. However, due to the relatively narrow spectral range of the 0.10 nm pulse, there are few spectral components distributed within the same band range between 1052.92 nm and 1052.95 nm. As a result, voltage-FBG1 is lower than voltage-FBG2 for this pulse, and the signal generator does not generate a trigger signal. Therefore, when the ambient temperature of FBG1 is set within the range of 25 °C to 27.5 °C, the failsafe system is capable of distinguishing between adequate PM states and inadequate PM states. However, as the ambient temperature of FBG1 rises to the range of 28 °C to 31 °C, the reflected band (1052.96~1052.99 nm) of FBG1 moves closer to the center band. The spectral energy of both the 0.15 nm and 0.10 nm pulses within this band exceeds that of the center band, leading to higher voltage-FBG1 compared to voltage-FBG2. In this case, the signal generator generates a trigger signal regardless of whether the pulse is broadened to 0.10 nm or 0.15 nm. Therefore, the failsafe system loses its ability to distinguish between the broadening states of the PM pulse within the temperature range of 28 °C to 31 °C. In summary, the failsafe system has an adjustable range of 2.5 °C to distinguish between the 0.15 nm spectral state and the 0.10 nm spectral state. The temperature control accuracy of TEC is 0.2 °C, providing a high redundancy to temperature drift within the failsafe system. Furthermore, we can adjust the reflected band of the FBG through TEC to distinguish different PM states. This method extends the application scope of the failsafe system.

The final aspect we explored was the impact of master oscillator (DFB) wavelength drift on spectral bandwidth discrimination. In the experiment, we precisely tuned the DFB wavelength within the range of 1052.96 nm to 1053.04 nm while keeping the ambient temperature of the FBGs constant. A 3.2 ns squared pulse with spectral widths of 0.15 nm and 0.10 nm was injected into the fiber front end. Figure 5b shows the response of the PM spectral failsafe system under different wavelength drifts of the DFB. Similar to Figure 5a, there were three distinct response regions in Figure 5b, including an indistinguishable, a distinguishable, and an unresponsive region, which are marked with light purple, light yellow, and light red, respectively. When the DFB wavelength drifted significantly in the short wave direction, that is, in the range of 1052.96~1052.985 nm, the failsafe system provided a 2.5 V trigger signal for both the 0.15 nm pulse and the 0.10 nm pulse. This wavelength, ranging from 1052.96 nm to 1052.985 nm, is called the indistinguishable region. When the DFB wavelength slightly deviated from 1053 nm, that is, between 1052.985 nm and 1053.015 nm, the failsafe system provided a trigger signal for the 0.15 nm pulse but not for the 0.10 nm pulse. In this case, the failsafe system could distinguish between an adequate PM state (0.15 nm) and an inadequate PM state (0.10 nm). This wavelength range from 1052.985 nm to 1053.015 nm is called the distinguishable region. When the DFB wavelength drifted significantly in the long wave direction, that is, in the range of 1053.015~1053.04 nm, the failsafe system did not generate a trigger signal for either the 0.15 nm pulse or the 0.10 nm pulse. This wavelength range from 1053.015 nm to 1053.04 nm is called the unresponsive region.

As shown in Figure 4 and Figure 5b, when the DFB wavelength shifts to the short-wave direction, the spectral energy reflected by FBG1 decreases slightly, while the spectral energy reflected by FBG2 increases slightly. Voltage-FBG1 is always greater than voltage-FBG2 for the 0.15 nm pulse in the wavelength range of 1053~1052.96 nm due to the voltage dividing effect of the RF compression circuit on the FBG2 branch. Then, the failsafe system provides a trigger signal. However, when the DFB wavelength is far from 1053 nm, that is, in the range of 1052.985 nm to 1052.96 nm, voltage-FBG1 is also greater than voltage-FBG2 for the 0.10 nm pulse. This is because the left peak sideband of the 0.10 nm pulse gradually falls within the reflection bandwidth of FBG1 as the wavelength offset exceeds 0.015 nm, leading to a sharp increase in the reflected energy. At this time, the failsafe system is unable to distinguish between the two PM states. Therefore, when the DFB wavelength shifts more than 0.015 nm towards the short-wave direction, the failsafe system enters the indistinguishable region. Similarly, as the DFB wavelength shifts towards the long-wave direction, the energy reflected by FBG1 decreases faster than that reflected by FBG2. When the DFB wavelength shift is less than 0.015 nm, that is, it is within the range of 1053~1053.015 nm, the reflected energy of both FBG1 and FBG2 changes smoothly. At this time, voltage-FBG1 is greater than voltage-FBG2 for the 0.15 nm pulse, voltage-FBG1 is smaller than voltage-FBG2 for the 0.10 nm pulse, and the failsafe system can output accurate trigger signals. Therefore, in the range of 1052.985~1053.015 nm, the failsafe system can distinguish the spectral broadening state of the PM light. However, as the DFB wavelength drifts towards the long-wave direction with a value greater than 0.015 nm, the energy reflected by FBG1 decreases sharply. As a result, voltage-FBG1 becomes less than voltage-FBG2 for both the 0.15 nm pulse and the 0.10 nm pulse, and the failsafe system will not output the trigger signal. Therefore, when the DFB wavelength shifts more than 0.015 nm in the long-wave direction, the failsafe system enters an unresponsive region. In summary, the failsafe system can correctly distinguish between adequate PM pulses and inadequate PM pulses within the wavelength range of 1052.985~1053.015 nm, with a tolerance of 0.03 nm. The wavelength stability of DFB lasers is typically less than 0.005 nm, providing a high degree of redundancy to the DFB wavelength drift.

The failsafe system utilizes two FBGs to compare the spectral energy of the sideband with that of the center band, thereby enabling the determination of the spectral broadening state. Compared to the single-FBG method, which only detects a PM sideband, the dual-FBG method has reduced dependence on the absolute energy of the pulse, demonstrating high reliability. Additionally, because only two FBGs are required, the failsafe system is more cost-effective than heterodyne detection. The failsafe system furthermore demonstrates robustness concerning pulse width, TEC temperature drift, and DFB wavelength drift. After long-term operation and testing, the failsafe system has shown high reliability with no failures and has been implemented in the fiber front end of the high-power laser facility. The design of the failsafe system based on dual gratings has advantages that include economy, durability, strong anti-interference abilities, and strong expansibility. Although the designed wavelength is 1053 nm, the failsafe system can be utilized for various high-power lasers in other bands by changing the FBGs. Furthermore, the concept of comparing sideband components with center band components instead of detecting absolute values provides valuable inspiration for the design of other detection systems.

## 5. Conclusions

In conclusion, we propose a dual FBG-based spectral failsafe system to indicate the presence or absence of adequate PM light, and we complete the design and performance characterization of the system. This method employs two temperature-sensitive gratings to extract the side band and center band components of FM light. Subsequently, the RF amplitudes of the two branches were compared after photoelectric conversion to determine whether the spectrum was broadened enough. The results showed that the PM spectral failsafe system can reliably detect laser pulses with widths greater than one modulation period (400 ps). These results demonstrate that the spectral failsafe system can reliably detect laser pulses for the temporally sculpted laser pulse with pulse widths greater than one modulation period (400 ps). However, for pulses with widths less than one modulation period, the initial phase of the phase modulation has a significant influence on the spectral shape, which may result in false judgments from the failsafe system. Furthermore, we studied the critical operating conditions of the failsafe system, mainly focusing on spectral bandwidth discrimination. The failsafe system has an adjustable temperature range of 2.5 °C, which is much higher than the TEC temperature control accuracy of 0.2 °C, allowing an adequate PM spectral state to be distinguished from an inadequate PM spectral state. Similarly, the failsafe system has a tolerance of 0.03 nm for wavelength shift, which is higher than the DFB wavelength stability of 0.005 nm. The failsafe system exhibits significant robustness against pulse width, TEC temperature drift, and DFB wavelength drift. The failsafe system utilizes an all-fiber configuration, eliminates the need for free-space coupling or alignment, and exhibits high reliability and compatibility, even under environmental perturbations. This attribute is highly valuable for the safe operation of high-power lasers and serves as a useful reference for the design of other detection systems.

## Figures and Tables

**Table 1:** Output characteristics of the PM spectral failsafe system under different pulse widths.

Pulse Width (ns)	PM		NO-PM	
	FBG1(V)	FBG2(V)	FBG1(V)	FBG2(V)
0.4	0.25	0.63	1.32	0
0.5	0.31	0.74	1.58	0
1.0	0.69	1.21	2.38	0
1.2	0.84	1.62	2.72	0
2.0	1.22	2.04	3.08	0
3.2	1.83	2.35	3.32	0
6.0	2.05	2.76	3.32	0
8.0	2.81	3.32	3.32	0
10.0 ^a^	3.32	3.32	3.32	0
21.8 ^b^	1.34	2.25	3.16	0

^a^ For 10 ns long square-wave pulses, the response voltage of PD is saturated due to high-energy injection. ^b^ The 21.8 ns pulse is an ignition-type temporally sculpted pulse, and its equivalent width is approximately 2.2 ns.

## Data Availability

Not applicable.
